# Vein of Galen aneurysmal malformation: does size affect outcome?

**DOI:** 10.1007/s00234-024-03347-6

**Published:** 2024-04-12

**Authors:** Costanza Parodi, Margherita Aluffi Valletti, Domenico Tortora, Silvia Buratti, Marisa Mallamaci, Giulia Tuo, Angela Pistorio, Andrea Moscatelli, Andrea Rossi, Mariasavina Severino, on behalf of the I. G. G. VGAM Team

**Affiliations:** 1 Neuroradiology Unit, IRCCS Istituto Giannina Gaslini, Via Gerolamo Gaslini 5, 16147 Genoa, Italy; 2 School of Medicine, University of Genoa, Genoa, Italy; 3 Neonatal and Pediatric Intensive Care Unit and Emergency Department, IRCCS Istituto Giannina Gaslini, Genoa, Italy; 4 Pediatric Cardiology and Cardiac Surgery Unit, Surgery Department, IRCCS Istituto Giannina Gaslini, Genoa, Italy; 5 Biostatistics Unit, Scientific Direction, IRCCS Istituto Giannina Gaslini, Genoa, Italy; 6 Department of Health Sciences (DISSAL), University of Genoa, Genoa, Italy

**Keywords:** Vein of Galen aneurysmal malformation, Semiautomated segmentation, Phase-contrast MR venography, Volume, Outcome

## Abstract

**Purpose:**

To validate a semiautomated method for segmenting vein of Galen aneurysmal malformations (VGAM) and to assess the relationship between VGAM volume and other angioarchitectural features, cardiological findings, and outcomes.

**Methods:**

In this retrospective study, we selected all subjects with VGAM admitted to the Gaslini Children’s Hospital between 2009 and 2022. Clinical data were retrieved from electronic charts. We compared 3D-Slicer segmented VGAM volumes obtained by two independent observers using phase-contrast MR venography to those obtained with manual measurements performed on T2-weighted images. The relationship between VGAM volumes and clinical and neuroimaging features was then explored.

**Results:**

Forty-three subjects with VGAM (22 males, mean age 6.56 days) were included in the study. Manual and semiautomated VGAM volumes were well correlated for both readers (*r* = 0.86 and 0.82, respectively). Regarding reproducibility, the inter-rater interclass correlation coefficients were 0.885 for the manual method and 0.992 for the semiautomated method (*p* < 0.001). The standard error for repeated measures was lower for the semiautomated method (0.04 versus 0.40 of manual method). Higher VGAM volume was associated with superior sagittal sinus narrowing, jugular bulb stenosis, and aqueductal stenosis (*p* < 0.05). A weak correlation was found between VGAM volume and straight sinus dilatation (*r* = 0.331) and superior sagittal sinus index (*r* =  − 0.325). No significant associations were found with cardiac findings, post-embolization complications, and outcome (*p* > 0.05).

**Conclusions:**

Semiautomated VGAM volumetry is feasible and reliable with improved reproducibility compared to the manual method. VGAM volume is not a prognostic factor for clinical outcome, but it is related to other venous findings with potential hemodynamic effects.

**Supplementary Information:**

The online version contains supplementary material available at 10.1007/s00234-024-03347-6.

## Introduction

The vein of Galen aneurysmal malformation (VGAM) is a rare congenital intra-arachnoidal and extra-cerebral vascular malformation, representing 30% of pediatric vascular and 1% of all pediatric congenital anomalies [1, 2]. VGAM is believed to arise at the choroid stage (8th–11th week of gestation) when choroidal arteries and their feeders become prominent in the cerebral vascular system. This malformation is the result of an abnormal embryonic development that leads to the shunting of arterial blood into the median prosencephalic vein of Markowski, a precursor of the vein of Galen. In these patients, the median prosencephalic vein of Markowski persists instead of undergoing a normal regression [3].

The clinical presentation of VGAM reflects its angio-architecture and shunt complexity as well as patient age. In neonates, the high-flow low-resistance lesion with direct arteriovenous connections leads to cardiac failure and respiratory insufficiency [4–6]. Later in infancy or childhood, patients may present with seizures, developmental delay, macrocephaly, and hydrocephalus [6, 7]. In adolescence and adulthood, symptoms comprise headaches, cognitive dysfunction, and, rarely, subarachnoid hemorrhage [6, 7].

Specialized intensive perinatal care and endovascular techniques are now available and have made VGAM a manageable condition [4, 8–10]. Research in the last few years has been focused on identifying early clinical and radiological prognostic features that might guide the clinical decision-making process. Several brain magnetic resonance imaging (MRI) findings have been evaluated, including (i) VGAM angioarchitectural features, (ii) arterial steal and/or pseudo-feeders, (iii) superior sagittal sinus stenosis, (iv) straight sinus dilatation, (v) VGAM pouch volume, (vi) posterior fossa dural sinus occlusion, and/or (vii) presence of brain lesions [11–18, 12, 17]. Among these, the most debated and controversial neuroimaging predictor is the volume of the venous pouch [12, 17, 19]. Historically, a large venous pouch accompanied by numerous feeders was associated with a worse prognosis [20, 21]. In 2017, Paladini et al. found that VGAM aneurysm volume > 20 cm^3^ was predictive of poor outcome and parenchymal injury [19]. However, according to recent studies, VGAM volumetry should no longer be considered a prognostic factor [12, 17].

The volume of the pouch is generally calculated from the three diameters (craniocaudal, laterolateral, and anteroposterior) using the ellipsoid formula:

$$V = 4\pi ABC/3$$

where *A* represents the height, *B* the width, and *C* the length of the pouch [12, 19].

Despite its wide use, we believe this application fails to represent the true morphology of most VGAM pouches, as it assumes a regular and edge-free shape.

Many segmentation algorithms and platforms are now easily accessible, even as open source, in the radiology field, and they are regularly used for evaluating other diseases. Studies on tumors have demonstrated that advanced segmentation techniques are highly performing and can automatically learn complex features representative of the heterogeneity of the tumor entity [22, 23]. Similarly, we hypothesize that segmentation techniques might reproduce more accurately the VGAM pouch, thus shedding light on the prognostic role of the VGAM pouch volume.

In the present study, we implemented a semiautomatic segmentation algorithm to define and calculate the VGAM pouch volume, and we compared the results of this method with those obtained using the ellipsoid formulas. We then assessed the relationship between the VGAM volume obtained with the new method and other neuroimaging and clinical features and explored the role of VGAM volume in the prediction of poor clinical outcome. As primary outcomes, we considered heart failure requiring endovascular treatment in the neonatal period. Secondary short-term outcomes included the presence of brain lesions at first MRI, the occurrence and severity of periprocedural neurologic complications, and mortality. Finally, the relationship between VGAM volume and neurological findings at last follow-up was explored.

## Methods

This single-center retrospective observational study has been approved by the Regional Ethical Committee (CER Liguria: 804/2021). Parental written informed consent was obtained for each exam. The study was conducted according to the Strengthening the Reporting of Observational Studies in Epidemiology (STROBE) guidelines [24].

### Patient recruitment

We recruited all newborns and infants with a diagnosis of VGAM admitted to the neonatal and pediatric intensive care unit of the Gaslini Children’s Hospital between October 2009 and April 2022. Inclusion criteria were defined as (i) the presence of a pre-operative MRI study performed at age < 1 year, (ii) a confirmed diagnosis of VGAM, (iii) presence of arterial and venous MR angiography (MRA), (iv) available data regarding clinical presentation and a preoperative echocardiogram, (v) available data on neurovascular intervention, and (vi) available outcome data in the neonatal period. Patients were excluded if MRI images presented motion artifacts.

### Brain MR imaging

Brain MR imaging was acquired under sedation using a neonatal 8-channel or 16-channel head coil on a 1.5 T magnet (Intera Achieva, Philips, Best, the Netherlands), or a 32-channel head coil on a 3 T magnet (Ingenia Cx, Philips, Best, the Netherlands).

The MRI protocol included a 2D or 3D T1 fast-field echo gradient–recalled, 2D T2-weighted images on three planes, axial T2* FFE or SWI, axial isotropic diffusion–weighted study (DWI and ADC), arterial and venous MR angiography using 3D time-of-flight (TOF) and phase contrast (PC) techniques, respectively, and non-invasive perfusion imaging using pulsed arterial spin labelling (pASL) at 1.5 T and 3D pseudo-continuous ASL (3DpCASL) at 3 T. Details of sequence acquisition parameters are provided in supplementary Table 1 (Online Resource 1).

### Qualitative and quantitative neuroradiological assessment

Brain MRI images were reviewed in consensus by two pediatric neuroradiologists (MS and DT) for the following angioarchitecture features: type of VGAM (choroidal versus mural), presence of superior sagittal sinus (SSS) and jugular bulb (JB) stenosis on venous MRA, and presence of small thalamic feeders and pseudo-feeders on both T2-weighted images and arterial MRA. Moreover, data on ventriculomegaly, aqueductal stenosis, and tonsillar caudal displacement were collected. Finally, the presence of white matter signal alterations, global brain atrophy, and ischemic/hemorrhagic lesions was noted. The latero-lateral diameter of the straight sinus at its narrowest point (SS-MD) and SSS index were calculated for each case as described in previous studies using the T2-weighted images [14, 17].

VGAM pouch volumes were obtained using the ellipsoid formula by two independent readers blinded for clinical outcome: a medical student with a 1-year experience in MR imaging (MAV, reader 1) and a neuroradiologist with a 15-year experience in pediatric neuroimaging (MS, reader 2). The volume was computed by measuring the maximum orthogonal diameters on the x, y, and z planes. These were measured on the patients’ 2D T2-weighted images using the computational tools provided by the Carestream PACS software package. The diameters were subsequently converted into radii and transcribed in the formula.

### Semiautomated segmentation of the VGAM pouch

**Fig. 1:**
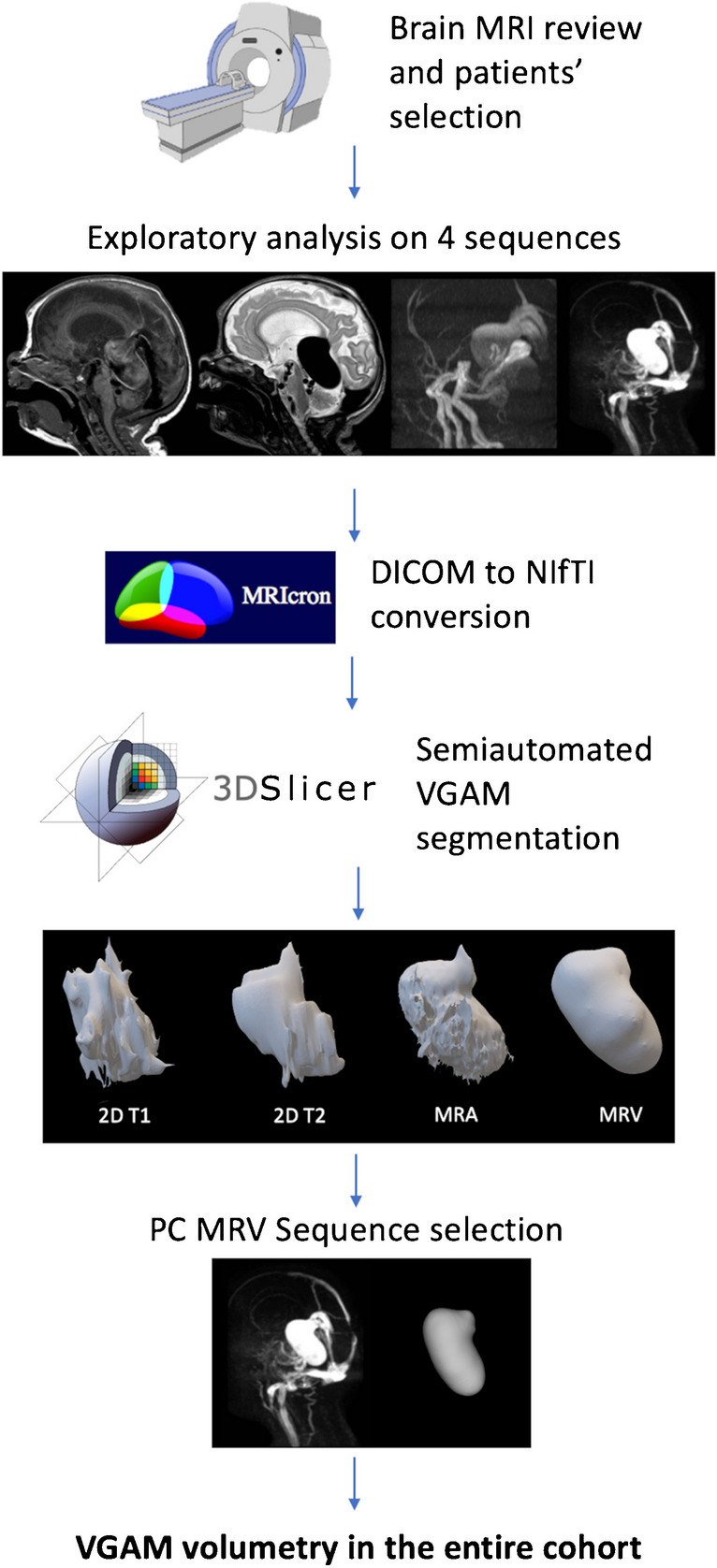
Study and segmentation workflow. After case selection (Sect. Introduction), the MRI images were retrieved from our PACS system and imported on the 3D Slicer software. A preliminary analysis on the sequence providing the highest quality 3D representation of the VGAM pouch was performed (Sect. Methods). The phase contrast venous MRA images obtained the highest scoring and were used to perform the VGAM pouch segmentation on the three planes in the entire cohort (Sect. Results). Figure created with BioRender.com

**Fig. 2:**
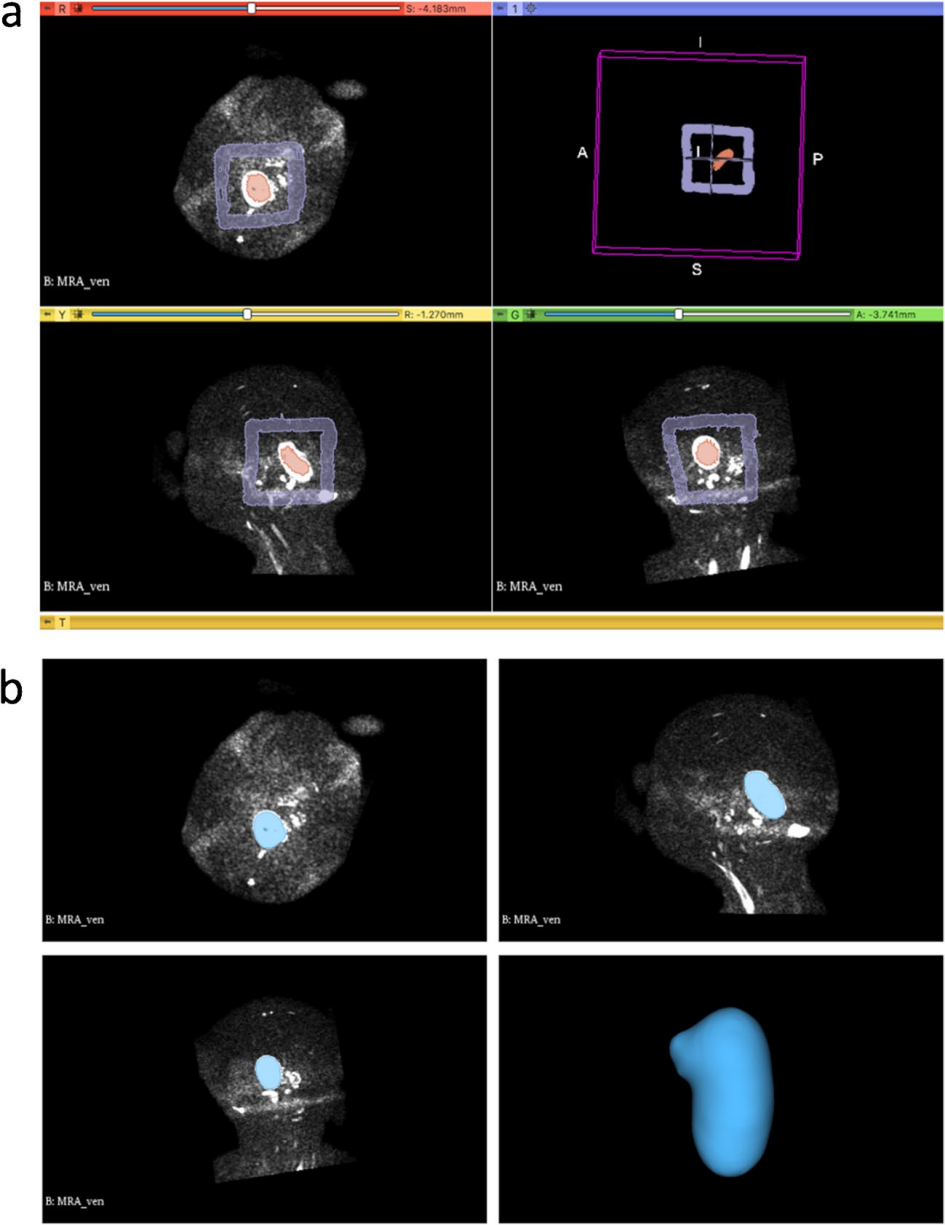
VGAM segmentation using the “Grow from seeds” algorithm from the Segment editor module of the 3D Slicer software. **a** The seed regions are drawn within (segment 1) and outside (segment 2) the VGAM pouch on the three planes with the function “paint.” **b** The “Grow from seeds” algorithm is initialized with automatic discrimination of the pouch from other brain structures

Figure 1 recapitulates the main steps of the semiautomated segmentation analysis. In detail, we first performed an exploratory analysis of multi-sequence MRI data in a subgroup of 10 subjects with VGAM (5 performed on a 1.5 T and 5 on a 3 T magnet) to select the MRI sequence that allowed the most accurate segmentation of the pouch. Images from 4 different MRI sequences, including 2D T2-weighted, 2D or 3D T1-weighted sequences, TOF arterial MRA, and phase-contrast venous MRA were converted with the open-source software MRIcron into a Neuroimaging Informatics Technology Initiative (NIfTI) format. The NIfTI images were then imported into the open-source 3D Slicer software by Brigham and Women’s Hospital (v. 4.10.2) that allows semiautomated segmentation and volume calculations [25]. Briefly, the process consisted of the following: (i) adding two segments in the Segment Editor module, (ii) drawing the seed regions within (segment 1) and outside (segment 2) the VGAM pouch on the three planes with the function “paint,” (iii) initializing the “Grow from seeds” algorithm for segment 2 which would automatically discriminate the pouch from other brain structures (Fig. 2), and (iv) manual refinement to verify that the segmentation followed the anatomical compartment boundaries properly. The VGAM volume was then extrapolated using the Segment Statistics function, and the three-dimensional (3D) segmentations were exported into a*.nnrd* file. The semiautomated segmentation method was implemented and validated by a bioinformatician (CP) with a 4-year experience in pediatric neuroimaging post-processing and analysis.

The VGAM segmentations obtained from the 4 sequences were independently assessed by two neuroradiologists (MS and DT, with 15 and 10 years’ expertise in pediatric neuroimaging, respectively) prior to any manual polishing, and assigned a score from 1 to 5, with 1 = Poor, 2 = Fair, 3 = Good, 4 = Very Good, and 5 = Excellent segmentation, as shown in the supplementary Fig. 1 (Online Resource 2).

All VGAM pouches were then independently segmented by the same readers who performed the linear manual measurements, i.e., reader 1 (MAV) and reader 2 (MS), using the sequence that rated the highest-quality scores. To avoid bias, a random patient was selected, and calculations for the volume were repeated 15 times for each approach by one reader.

### Clinical, cardiac, and neurological assessment

Clinical data were retrieved for all subjects from the electronic clinical charts of the patients by two experienced pediatric intensive care specialists (SB and MM). Preoperative echocardiographic examinations were reviewed by an expert pediatric cardiologist (GT) assessing for the following parameters: right end-diastolic diameters (REDD), left ventricular fractional shortening (LV-FS), estimation of pulmonary hypertension (PH) by means of PH index (ratio between systolic pulmonary artery pressure and systemic arterial pressure), flattening of the interventricular septum (IVS), ductus arteriosus blood flow direction, and reversal flow at the level of the aortic isthmus. The shape of the IVS was described based on the right-to-left side motion as normal, intermediate, and complete right-to-left shift with left ventricular collapse [26].

Heart failure was defined by the presence of tachycardia, signs of respiratory distress, and clinical and biochemical markers of poor organ perfusion (oliguria and lactic acidosis). Longitudinal clinical observation after birth and progressive implementation of medical therapy and respiratory support led to the definition of heart failure. We categorized patients as neonates at risk (NAR) if they needed intensive care support for their cardiopulmonary failure (inotropes and invasive ventilation) and thus underwent urgent neonatal embolization. Minor embolization complications were defined as ischemic and/or hemorrhagic lesions with no or mild signs of neurological dysfunction. Major complications were defined as lesions associated with seizures and/or major neurological dysfunction. Causes of death were reported as endovascular procedure complication, heart failure with multiorgan failure, or palliation for severe brain damage.

Detailed neurological examinations were conducted at last follow-up by an experienced pediatric neurologist (GG). The level of the child’s functional ability was scored using the Pediatric Overall Performance Category (POPC scale), with the following scores: 1, normal life; 2, mild disability; 3 and 4, moderate and severe disability; 5, coma/vegetative state; and 6, brain death, as detailed in the Supplementary Table 2 (Online Resource 3) [27].

### Intervention: therapy and embolization

Data regarding the embolization procedures and therapy were obtained from the clinical charts and by reviewing the digital subtraction angiography (DSA) images. Briefly, the procedure aimed to progressively occlude VGAM arterial feeders using *N*-butyl-2-cyanoacrylate (NBCA) or other liquid embolic systems. The transarterial route was preferred, by catheterizing the femoral artery with a catheter ranging from 1.2 to 1.5 French. A multiple-staged embolization, performed with a mixture of NBCA and contrast bolus, was used to minimize the risk of rapid venous thrombosis and intracerebral hemorrhage. The main aim of the first neonatal procedure was to reduce the shunt by at least 30%, to arrest or slow down the progression of cardiac insufficiency. The procedure was not performed in the presence of severe brain damage or in case of spontaneous VGAM thrombosis.

Epinephrine, milrinone, and levosimendan were the medications commonly used to treat the patients. The dosage of inotropic drugs was recorded, and scores were computed. The inotropic score (IS) and vasoactive inotropic score (VIS) were calculated as described in previous studies [28].

### Statistical analysis

Clinical and neuroradiological data were reported as means and standard deviations or/and as frequencies depending on the nature of the variable. In the preliminary analysis, we measured inter-rater reliability regarding the choice of sequence for the semiautomated segmentation using weighted Cohen’s K statistics. The sequence providing the best segmentation results was selected if a substantial agreement between raters was shown (Cohen’s K > 0.6).

The volumes obtained with the ellipsoid formula and the semiautomated segmentation method were then compared by testing for validity and reproducibility. Validity was evaluated using Spearman’s rank correlation and Bland–Altman analyses for both readers. Bias, upper and lower bounds were defined, respectively, as the average volume difference between the two approaches and bias $\pm$ 1.96 × standard deviation.

Intraclass correlation coefficients (ICC) using a two-way mixed model were used to assess the reproducibility of the two methods for both readers. Based on 95% confidence intervals of the ICC estimate, values less than 0.5, between 0.5 and 0.75, between 0.75 and 0.9, and greater than 0.9 were indicative of poor, moderate, good, and excellent reliability, respectively [29].

The standard error of measurement was then calculated on the multiple measurements and segmentations performed by one reader.

Further analysis was performed on the volume data obtained with the semiautomated segmentation to avoid violation of the independence of observations, outliers, and multicollinearity. The Mann–Whitney *U* test was performed to measure statistical significance with dichotomous clinical variables, while the Kruskal–Wallis *H* was used when the clinical variable included more than 2 groups. Correlation was measured between the VGAM volume and other continuous variables by performing a Spearman’s rank coefficient test, based on rejection of the normality of distribution.

To compare the contribution of each individual parameters in predicting the outcome, all variables were finally used in a discriminant function analysis, in which a categorical dependent grouping variable is determined from more independent predictor variables. For each parameter, a standardized coefficient indicates the unique contribution of each predictor variable to the function. The magnitudes of these coefficients indicate how strongly the predictor variables affect the dependent variable.

The level of significance was set at 0.05. All statistical analyses were performed using the SPSS Statistics software, v21 (IBM, Armonk, NY), and verified by a senior biostatistician (AP).

## Results

### Clinical and neuroradiological data

Between 2009 and 2022, 49 patients with VGAM were admitted to the Gaslini Children’s Hospital. One neonate died before undergoing a brain MRI, three subjects had an incomplete brain MRI protocol, and 2 patients had a poor-quality MR scan. In total, 43 patients with VGAM were included in this study (22 males, mean age at first MRI 6.56 days, range 0–99 days). Of these, 39 subjects were enrolled during the neonatal period. Patients’ characteristics are summarized in Table 1. Twenty-two neonates (51%) underwent the first MRI examination on a 1.5 Tesla scanner and 21 (49%) on a 3 Tesla scanner. Table 2 reports the results of the qualitative analysis of the brain MRI studies of the patients.

**Table 1:** Demographic and general features of the patients

**Patients’ characteristics**	
Sex, M (%)	22 (51.1)
Mean GA at birth (range)	38 w (30–41)
Mean age at first MRI (range)	6.56 d (1–99 d)
Mean age at embolization (range)	38.74 d (2–198 d)
Mean age at last follow-up (range)	5.49 y (2 d–14 y)
Embolization (%)	35 (81)
Neonatal embolization (%)*†	22 (63)
Embolization complications (%)†	15 (43)
Minor complications (%)†	7 (20)
Major complications (%)†	8 (23)
Palliated (%)	3 (7.6)

*d* days, *GA* gestational age, *M* males, *MRI* magnetic resonance imaging, *w* weeks, *y* years

^*^Treatment performed in 17 cases for hemodynamic reasons and in 5 cases for neuroradiological features including pseudo-feeders

^†^Data available on the 35 embolized patients

**Table 2:** Qualitative neuroradiological findings of the patients

	Yes (%)	No (%)
Brain MRI features (*n* = 43)		
Ventriculomegaly	14 (33)	29 (67)
SSS stenosis	25 (58)	18 (42)
JB stenosis	24 (53)	19 (47)
ICV drainage into VGAM	9 (21)	34 (79)
Small thalamic feeders	16 (37)	27 (63)
Pseudo-feeders	17 (40)	26 (60)
Global brain atrophy	3 (7)	40 (93)
Ischemic/hemorrhagic lesions	13 (30)	30 (70)
WM signal alterations	18 (42)	25 (58)
Tonsillar herniation	0 (0)	43 (100)
Aqueductal stenosis	25 (58)	18 (42)

*ICV* internal cerebral veins, *JB* jugular bulb, *MRI* magnetic resonance imaging, *RV* right ventricle, *SSS* superior sagittal sinus, *WM* white matter

### VGAM volume analysis

The sequence achieving the best scoring in the preliminary analysis was the phase contrast MR venography, with a mean score of 4.5 and 4.6 for readers 1 and 2, respectively, corresponding to an excellent segmentation with a weighted Cohen K of 0.8.

**Fig. 3:**
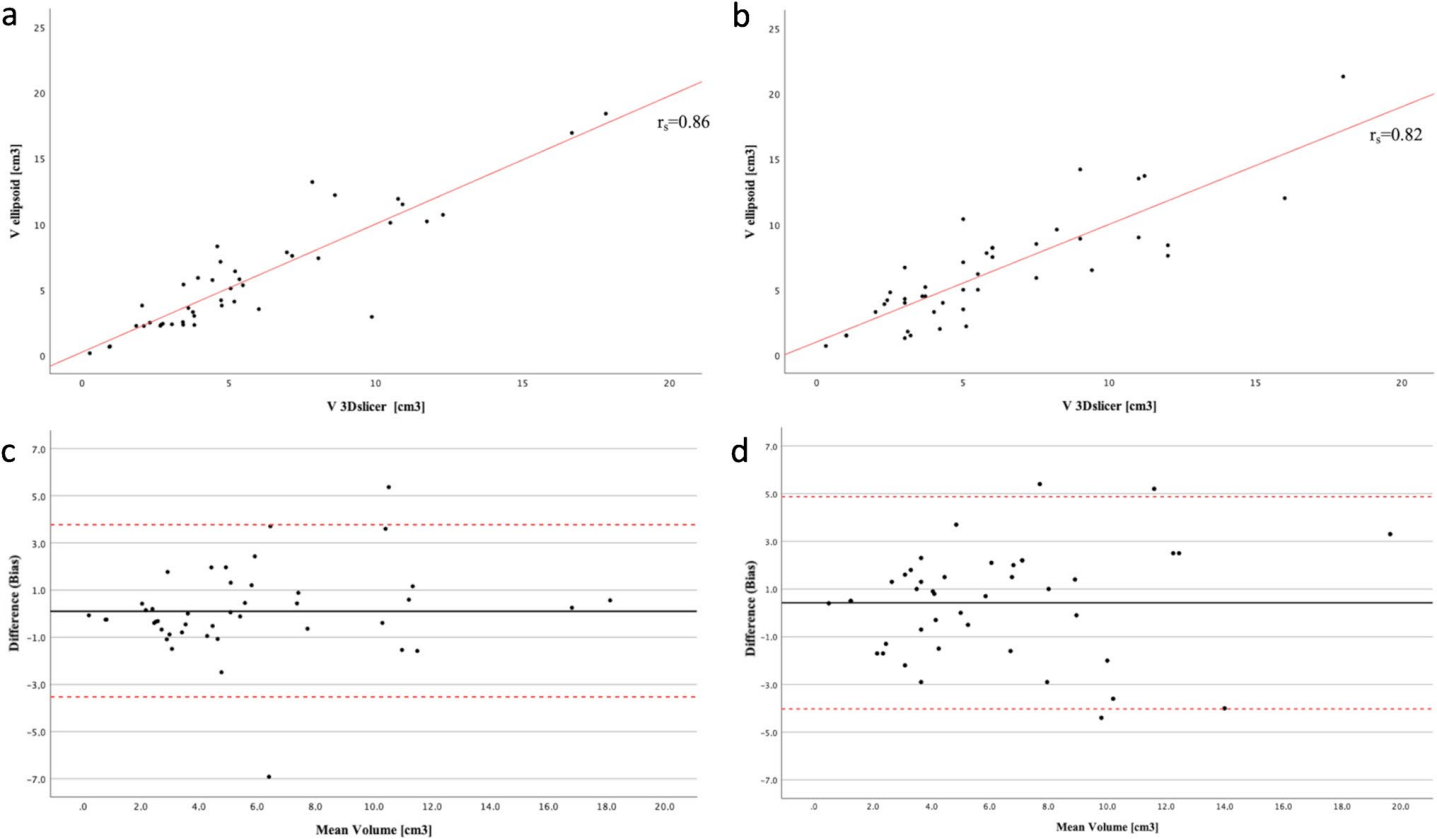
Scattered and Bland–Altman plots showing the agreement between the two methods for evaluating the VGAM volume. **a**, **b** Scattered plots showing good correlation between the two methods both for reader 1 (**a**) and reader 2 (**b**). **c**, **d** Bland–Altman plots reveal that the bias between manual and semiautomated methods was very small both for reader 1 (**c**) and reader 2 (**d**), i.e., 0.1 and 0.4, respectively. Around 5% of the points were lying outside the limits, specifically 2/43 (4%) for reader 1 and 3/43 (6%) confirming a good agreement between the approaches. Bias is the mean difference between the volumes of the Slicer 3D and ellipsoid (black line). Red dashed lines represent the upper and lower limits of agreement ($\pm 1.96\sigma$)

Table 3 reports the median VGAM volumes obtained with the manual and semiautomated method for both readers. The volumes measured by the two methods correlated well, with *r*_s_ (41) = 0.86, *p* < 0.001 for reader 1 and *r*_s_ (41) = 0.82, *p* < 0.001 for reader 2 (Fig. 3a, b). Bland–Altman analysis showed that the bias between manual and semiautomated methods was very small for both readers, i.e., 0.1 for reader 1 and 0.4 for reader 2 (Fig. 3c, d). Around 5% of the points were lying outside the limits, specifically 2/43 (4%) for reader 1 and 3/43 (6%) confirming a good agreement between the approaches.

**Table 3:** VGAM volume calculated with the semiautomatic method and with the ellipsoid formula for both readers

VGAM volume (cm^3^)	*R*1 med (IQR)	*R*1 (min–max)	*R*2 med (IQR)	*R*2 (min–max)
3D Slicer	4.7 (3.24–7.49)	0.25–17.84	5 (3.01–8.21)	0.3–18.2
Ellipsoid formula	4.2 (2.46–7.71)	0.18–18.40	5.2 (3.51–8.42)	0.7–21.33

*IQR* interquartile range, *Max* maximal, *Med* median, *Min* minimum, *R1* reader 1, *R2* reader 2, *VGAM* vein of Galen malformation

Regarding reproducibility, the inter-rater ICC was 0.885 (confidence interval 0.797–0.936) for the manual method and 0.992 (confidence interval 0.996–0.998) for the semiautomated method (*p* < 0.001).

Repeated measures on the same subject showed less variability using 3D Slicer compared to the ellipsoid formula with a standard error of 0.04 cm^3^ (median 11.94, IQR 11.78–11.98 cm^3^) and 0.40 cm^3^ (median 11.69, IQR 10.99–12.99 cm^3^), respectively, as shown in the Supplementary Fig. 2 (Online Resource 3).

### Associations between VGAM volume and clinico-instrumental features

Table 4 demonstrates the relationship between the VGAM volume obtained with 3D slicer and qualitative neuroimaging features. We observed a higher volume of the pouch in patients with SSS stenosis (5.20 cm^3^; IQR 3.60–10.62 versus 4.02 cm^3^; IQR 2.23–5.38), JB stenosis (5.11 cm^3^, IQR 3.77–10.69 versus 3.61 cm^3^, IQR 2.30–5.47), and aqueductal stenosis (4.79 cm^3^, IQR 3.43–10.62 versus 3.81 cm^3^, IQR 1.98–5.38) with *p*-values < 0.05.

**Table 4:** Relationship between VGAM volume and other neuroradiological features

			***V***_**3D SLICER**_	
		*n* (%)	Med (IQR)	*p*-value
Brain MRI features (*n* = 43)				
VGAM type	Choroidal	36 (84)	4.51 (3.33–7.89)	0.910
	Mural	7 (16)	5.05 (2.83–6.57)	
Ventriculomegaly		14 (33)	4.70 (3.76–8.61)	0.140
SSS stenosis		25 (58)	5.20 (3.60–10.62)	**0.036**
JB stenosis		24 (56)	5.11 (3.77–10.69)	**0.045**
ICV drainage into VGAM		9 (21)	4.43 (2.24–6.44)	0.418
Small thalamic feeders		16 (37)	3.87 (2.23–6.52)	0.167
Pseudo-feeders		17 (40)	5.17 (3.60–10.62)	0.224
Aqueductal stenosis		25 (58)	4.79 (3.43–10.62)	**0.044**

*ICV* internal cerebral vein, *IQR* interquartile range, *JB* jugular bulb, *MRI* magnetic resonance imaging, *SSS* superior sagittal sinus, *V* volume, *VGAM* vein of Galen malformation, *WM* white matter

*p*-values < 0.05

**Fig. 4:**
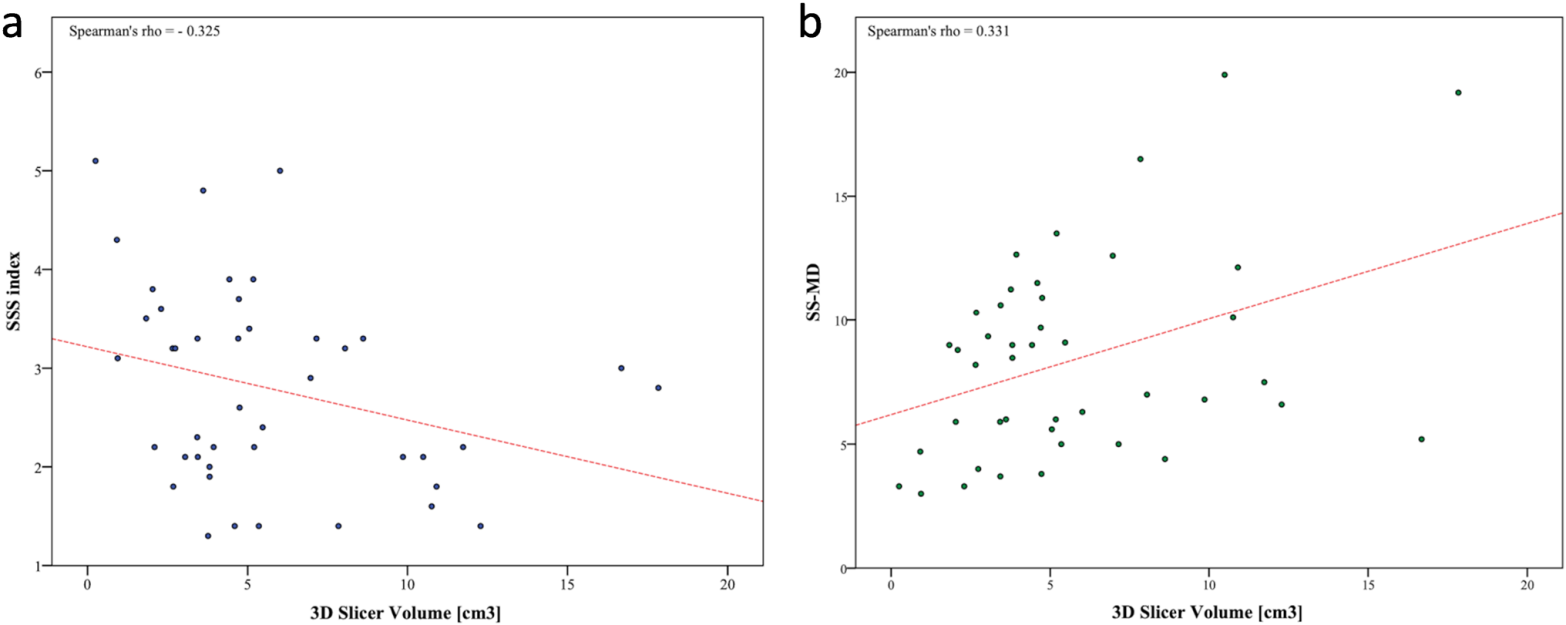
Scatterplot graphs showing the correlation between the VGAM volume and quantitative neuroradiological features for both methods. **a** Correlation with the superior sagittal sinus (SSS) index. **b** Correlation with the latero-lateral diameter at the narrowest point of the straight sinus (SS-MD)

Regarding the quantitative parameters, a weak positive correlation was measured between VGAM volume and SS-MD (rho = 0.331) whereas a weak negative correlation was observed for the SSS index (rho =  − 0.325) (Fig. 4).

Functional and anatomical cardiac features at birth were available for 39 neonates with VGAM (Table 5). No significant differences were found regarding VGAM volume, and the null hypothesis was accepted for all parameters (*p* > 0.05).

**Table 5:** Relationship between VGAM volume and cardiological features at birth

		*n* (%)	***V***_**3D SLICER**_	
			Med (IQR)	*p*-value
Neonatal cardiac US (*n* = 39)				
RV dilation	Yes	24 (61.5)	4.70 (3.43–8.04)	0.529
Shunt in DA	L to R	11 (28.2)	4.90 (2.91–9.18)	0.137
	L = R	13 (33.3)	4.10 (2.48–5.44)	
	R to L	15 (38.4)	5.20 (3.81–10.76)	
Shape IVS	Normal	14 (35.8)	3.43 (2.34–6.58)	0.268
	Intermediate	19 (48.7)	4.89 (3.53–10.02)	
	R to L	6 (15.3)	6.08 (3.89–8.56)	
PH index	> 1	14 (37)	4.95 (3.58–10.56)	0.469
	1	10 (25.6)	5.47 (3.62–10.80)	
	< 1	15 (38.4)	4.75 (2.74–6.01)	

*DA* ductus arteriosus, *IQR* interquartile range, *IQR* interquartile range, *IVS* interventricular septum, *L* left, *Med* median, *PH* pulmonary hypertension, *R* right, *RV* right ventricular, *US* ultrasound, *V* volume

The discriminant function analysis demonstrated a greater contribution of SSS stenosis (standardized coefficient of 1.33), JB stenosis (standardized coefficient of 1.02), SS-MD (standardized coefficient of 0.16), and SSS index (standardized coefficient of 0.08) in predicting NAR when compared with VGAM volume (standardized coefficient of 0.02).

### VGAM volume and outcomes

Table 6 reports the relationship between the VGAM volume and the short- and long-term outcome measures. Heart failure occurred in 20/39 neonates (51.2%), of whom 17 (85%) were categorized as NAR due to high-output heart failure requiring EVT in the neonatal period and 3 were palliated due to the severe brain damage at birth (these 3 patients were not included in the analysis). We did not find significant differences between the VGAM volume and presence of heart failure at birth or in the NAR subgroup. Similarly, no differences in VGAM volume emerged for death (*p*-value > 0.05).

**Table 6:** Relationship between VGAM volume and outcomes and embolization complications

			***V***_**3D SLICER**_	
		***n* (%)**	**Med (IQR)**	***p*-value**
Short-term outcomes				
Heart failure (*n* = 39)*		20 (51)	5.19 (3.81–10.69)	0.120
NAR (*n* = 36)^§^		17 (44)	5.17 (3.79–9.30)	0.208
Deaths (*n* = 43)		7 (16)	5.17 (3.81–10.49)	0.323
I/H lesions at first MRI (*n* = 43)		13 (30)	4.70 (3.60–10.70)	0.425
WM signal alterations (*n* = 43)		18 (42)	4.88 (3.34–10.59)	0.176
Global brain atrophy (*n* = 43)		3 (7)	10.49 (2.66–17.84)	0.321
Long-term outcomes				
POPC score (*n* = 36)^	1	26 (60)	4.71 (2.57–7.32)	0.337
	2	3 (7)	3.94 (3.76–4.43)	
	3	2 (5)	12.40 (n.a.)	
	4	5 (12)	3.44 (2.38–12.01)	
Epilepsy (*n* = 36)^		8 (22)	5.36 (2.87–12.15)	0.421
Treatment				
Embolization complications (*n* = 35)		17 (48.5)	5.05 (3.81–10.49)	0.657

*EVT* endovascular treatment, *I/H* ischemic/hemorrhagic, *IQR* interquartile range, *n.a.* not applicable, *NAR* neonates at risk, *Med* median, *MRI* magnetic resonance imaging, *POPC* Pediatric Overall Performance Category, *WM* white matter

^*^Analysis performed in the 39 subjects presenting in the neonatal period

^§^Three neonates who died after palliation for severe brain lesions were excluded from the analysis

^Analysis performed in the 36 surviving subjects

At first MRI, 13/43 (30%) of subjects had small-to-medium sized ischemic and/or hemorrhagic lesions, 18/43 (42%) had white matter signal alterations, and 3/43 (7%) showed global brain atrophy. None of these features was associated with larger VGAM volumes (*p*-value > 0.05).

At last follow-up, 60% (26/43) of our cohort was assigned a POPC1, 7% (3/43) a POPC2, only 5% (2/43) scored a POPC3, and 12% a POPC4. A total of 36 cases had EEG data available, among whom 8 were diagnosed with epilepsy (22%). No associations were observed between the VGAM volume and epilepsy or POPC score (*p*-value > 0.05).

### Treatment and intervention strategies

Table 7 reports the median and IQR values for the IS and VIS scores in the 39 neonates with VGAM. No correlation was observed between the IS and VIS scores and the VGAM volume (*p*-values > 0.05).

**Table 7:** Relationship between VGAM volume and pharmacological cardiovascular support

	Median (min–max)	[IQR]	*V*_3D SLICER_ *p*-value
Pharmacological support (*n* = 39)			
IS score	0 (0–20)	[0–10]	0.226
VIS score	4 (0–24)	[0–6]	0.187

*IQR* interquartile range, *IS* inotrope score, *Max* maximal, *Min* minimum, *V* volume, *VIS* vasoactive inotrope score

In the whole cohort, endovascular staged embolization was performed in 35/43 subjects (81%) with a median age at first embolization of 33.2 days (range 2 days–6 months). A total of 22/35 cases underwent endovascular treatment in the neonatal period: 17 for severe heart failure and 5 for the presence of neuroradiological risk factors, such as pseudofeeders, even in the absence of severe heart failure [12]. Multiple embolization procedures were completed in 19/35 (54%) patients (range of procedures, 2 to 9) after multidisciplinary team evaluations. After embolization, 15/35 (43%) subjects developed periprocedural neurological complications (8 major and 7 minor). In four patients (4/43, 10%) who were asymptomatic at birth and clinically stable, the VGAM underwent spontaneous thrombosis and was not treated.

No association was observed between endovascular procedure complications or the IS and VIS score and the VGAM volume (Table 6 and 7) (*p*-value > 0.05).

## Discussion

In the present study, we demonstrated that quantitative semiautomated VGAM volumetry is feasible with reliable and reproducible results as compared to linear measurements. Indeed, only a few cases were lying outside of the agreement limits between the two methods, showing that the volumes calculated with the semiautomated approach do not diverge significatively from those obtained using the ellipsoid formula. Post hoc inspection of the segmented boundaries suggested that the outliers in variability were mainly due to judgment calls of the operator. These cases had large transitions between the VGAM aneurysm and the straight sinus, thus not enabling a clear separation of these anatomical structures and requiring operator judgment on where to draw the border between the two. The narrower confidence interval by the semiautomated method suggested a better reproducibility compared to the manual method. Of note, the standard error in repeated measurement was found to be lower in the *V*_3D SLICER_ than the *V*_ELLIPSOID_ (0.04 versus 0.40), indicating less variability results from the 3D Slicer method. Of note, phase-contrast MR venography provided the best segmentation results among all sequences. While arterial TOF angiography better depicts the VGAM arterial feeders [11], phase-contrast MR venography provides better contour delineation with high signal intensity of the VGAM pouch and is ideal to obtain VGAM volumetry. Interestingly, this sequence has also been used to obtain quantitative measurements of flow inside the VGAM pouch [30, 31], thus providing interesting insights regarding the flow dynamics of the shunt. A traditional drawback associated with phase-contrast imaging is the acquisition time penalty [32]. However, the use of parallel imaging and accelerating techniques has led to significantly reduced times to acquire phase-contrast MR angiograms [32].

Regarding the relationship with other neuroradiological features, we observed larger VGAM volumes in subjects with SSS narrowing, JB stenosis, and aqueductal stenosis. While the latter feature is explained by the VGAM mass effect, the relation with SSS and JB stenosis is more complex and likely relies on hemodynamic mechanisms. SSS narrowing is associated with poor clinical outcome in patients with VGAM [14] but its etiology is not well understood. The main draining route of the VGAM is toward the straight sinus or through a persistent falcine sinus when the straight sinus is absent or small [20]. Saliou et al. proposed that a decrease in the SSS diameter could reflect a reduction of venous flow through the cortical veins caused by the shunt, associated with venous compression due to high intracranial pressure [14]. We thus hypothesize that the larger VGAM size might be related to increased arterial steal towards the shunt and higher intracranial pressure, with consequent venous diameter reduction of the SSS. Future studies using noninvasive MR techniques to assess hemodynamics within the VGAM pouch, such as 4-Dimensional Flow Magnetic Resonance Imaging [31], are awaited to clarify whether larger VGAM are characterized by higher blood flow and/or pressure gradients through the shunt.

JB stenosis or occlusion is another prognostic factor associated with poor prognosis due to worsening of cerebral venous hypertension [13, 14]. JB stenosis is rarely present at birth and usually develops later during the disease course, compromising venous outflow and causing complications related to chronic venous hypertension [13]. The underlying pathophysiology is still unknown, but several hypotheses have been formulated, including intrinsic “dysmaturative” vessel factors or extrinsic “hemodynamic” mechanisms [4, 14, 33, 34]. According to the latter, JB stenosis might protect the heart by decreasing the venous pressure arising in the right heart due to the high-flow shunt [14]. A recent study using phase-contrast MR sequences with arterial and venous dual velocity encoding showed a high-complexity recirculating flow pattern in the venous system of infants with VGAM, thus supporting the “hemodynamic” theory [35]. We speculate that, as for the SSS narrowing, larger VGAM are related to more complex flow patterns and consequent hemodynamic changes in the venous system. The integration of VGAM volumetry with computational fluid dynamics in prospective cohorts might shed light on this issue.

Finally, we found a positive correlation between the VGAM volume and SS-MD, a well-known neonatal prognostic factor in fetuses and neonates with VGAM [17, 19]. In 2017, Paladini et al. first reported a poor clinical prognosis, including neurologic sequelae, neonatal death, and termination of pregnancy, in fetuses with VGAM and straight sinus dilatation [19]. Subsequently, Arko et al. confirmed that dilatation of the straight sinus at the point of greatest constriction to flow return from the malformation to the systemic circulation was sharply predictive of mortality and the need for neonatal intervention [17]. In particular, they found that a SS-MD of > 6.2 mm in the neonatal MRI and/or > 5.2 mm in the fetal MRI strongly predicted clinical evolution to heart failure requiring early endovascular treatment [17]. Despite the underlying mechanism remaining unknown, higher venous pressure has been found in patients with an enlarged straight or falcine sinus, likely transmitting the intra-aneurysmal pressure to the general cerebral venous system [36]. Interestingly, Quisling and Mickle also showed a positive relationship between VGAM size and venous pressure, with larger aneurysms presenting higher venous pressures [36], thus supporting our findings.

Using the VGAM volumetry obtained with this new semiautomated quantitative method, we did not find any association with anatomical and functional cardiac features or outcome measures at birth. Moreover, the discriminant function analysis confirmed that VGAM volume has a marginal role in predicting the NAR outcome as compared to other well-known neuroimaging findings (i.e., JB and SSS stenosis and straight sinus dilatation). These findings further support recent evidence that median prosencephalic varix size itself is not a predictor of clinical evolution to heart failure requiring early endovascular intervention or death in the neonatal period [12, 17]. As such, the relationship between VGAM volume and the other venous predicting factors is independent from the outcome and likely due to different hemodynamic factors. Also, no relationship was noted between VGAM volumetry and either the dosage of inotropic drugs required to maintain clinical stability in the neonatal period or the occurrence of post-embolization complications. In addition, in contrast with the study of Paladini et al. [19], VGAM volume was not predictive of brain damage, including ischemic/hemorrhagic lesions, white matter signal alterations, and global brain atrophy. Finally, despite the presence of possible bias due to different intervening factors, we did not find a relationship between the VGAM volume and long-term outcome, including levels of functional ability and disability and the presence of epilepsy at last follow-up.

The major limitations in this study are the retrospective design and small sample size, in keeping with the rarity of the disorder. Regarding the volumetric analysis, in some cases, there was a discrepancy between the results obtained with the linear measurements and semiautomated method due to anatomical reasons, underlying the necessity of manual refinement to draw the lesion boundary in complex cases. However, in our opinion, the reduction of operator variability and the relative simplicity of the method outweigh this problem. Among other limitations, we did not use digital subtraction angiography to confirm the data obtained from brain MRI and MRA; indeed, angiography was performed in newborns only for therapeutic purposes, using a limited amount of contrast for diagnostic runs due to the small age and weight of the patient. As such, full replenishment of the pouch was purposedly not achieved in most cases. Moreover, the positioning of the head during angiography often leads to oblique sagittal and coronal plane acquisitions, hindering precise measurements. Conversely, our MRI protocols were performed with strict rules for the alignment of the sagittal (with midline planes always including both the cerebral aqueduct and pituitary stalk), coronal (oriented along the floor of the fourth ventricle), and axial sequences (aligned along the bi-commissural plane). Notably, most of our cases were treated with a biplanar angiography suite in which 3D rotational acquisitions were not technically available, representing a further limitation against the possibility of obtaining multiplanar reformatting.

Finally, there is a possible influence of changes in neurointerventional devices on the long-term outcome during the wide interval time of the study. Indeed, it is possible that the post-embolization complication rates have been reducing over the years with better clinical outcome, thanks to increased operator experience and skills, improved patient selection, and better devices and technology. However, as reported by Brinjiki et al. in their systematic review and metanalysis [37], there is not enough data to determine clinical and angiographic outcomes by type of embolic agent or device used, and it is thus difficult to sort out short- and long-term morbidity and mortality related to the endovascular treatment. Recently, Bathia et al. found that using a microcatheter with a distal outer diameter of more than 2.0 Fr was a significant predictor of poor neurological outcomes [38]. More studies are needed to understand the real impact of devices and technology on the long-term outcome of the patients with VGAM.

## Conclusions

In summary, semiautomated segmentation and VGAM volumetry are feasible with low variability and better representation of the aneurysmal shape compared to linear measurements. This 3D tool is easy to use and may be particularly useful in research settings. We confirmed that VGAM volume is not an independent predictor of short- and long-term outcome, but rather an imaging correlate of the associated venous abnormalities, such as the SSS narrowing, JB stenosis, and straight sinus dilatation. Future prospective studies on larger cohorts are needed to validate this approach and to shed light on the relationship between the aneurysm volume and VGAM hemodynamics.

### Supplementary Information

Below is the link to the electronic supplementary material.

Supplementary file1 (PDF 1108 KB)
