# Supplementary material for: Vein of Galen aneurysmal malformation: does size affect outcome?
Source: Neuroradiology. 2024 Apr 12;66(6):1031–42. doi: 10.1007/s00234-024-03347-6 (PMC11133167; doi:10.1007/s00234-024-03347-6)
Supplement: Supplementary file 1 — Supplementary file1 (PDF 1108 KB) [file 234_2024_3347_MOESM1_ESM.pdf]

Online Resource 1

Supplementary Table 1. Magnetic resonance imaging parameters for the study protocol

	**T1-weighted imaging**				**T2-weighted imaging**		**Phase-contrast venous MRA**		**TOF arterial MRA**	
**Acquisition type**	2D		3D		2D		3D		3D	
**Manufacturer**	Philips		Philips		Philips		Philips		Philips	
**Tesla**	1.5	3	1.5	3	1.5	3	1.5	3	1.5	3
**Repetition time**	800	700	420	600	6000	6000	14	11	17	23
**Echo time**	12	14	18	25	140	160	6.1	7	6.9	4
**Flip angle**	70	70	80	90	90	90	10	10	20	18
**Slice thickness**	4	2	1	1	3	2	1.6	1.6	1.4	1.2
**Acquisition matrix**	230 x 163	200 x 160	256 x 256	196 x 196	512 x 512	360 x 289	144 x 109	208 x 156	268 x 207	400 x 250
**Voxel dimension**	1	1	1	1	0.4	0.4	1.4	0.9	0.6	0.3
**Spacing between slices**	3.3	2.2	1	0.5	3.3	2.2	0.8	0.8	0.7	0.6
**Other**							PC velocity 15cm/s	PC velocity 13cm/s		

Legend: MRA, Magnetic resonance angiography; PC, phase contrast

Online Resource 2

Supplementary Figure 1. 3D slicer segmentation of the VGAM pouch using four MRI sequences of a neonatal case with VGAM. Sagittal T1-weighted (a), T2-weighted (b), arterial TOF MR angiography (c) and venous phase-contrast MR angiography (d) images with corresponding 3D segmentation of the VGAM pouch (e-h). Note that the best results are provided by the venous MRA images.

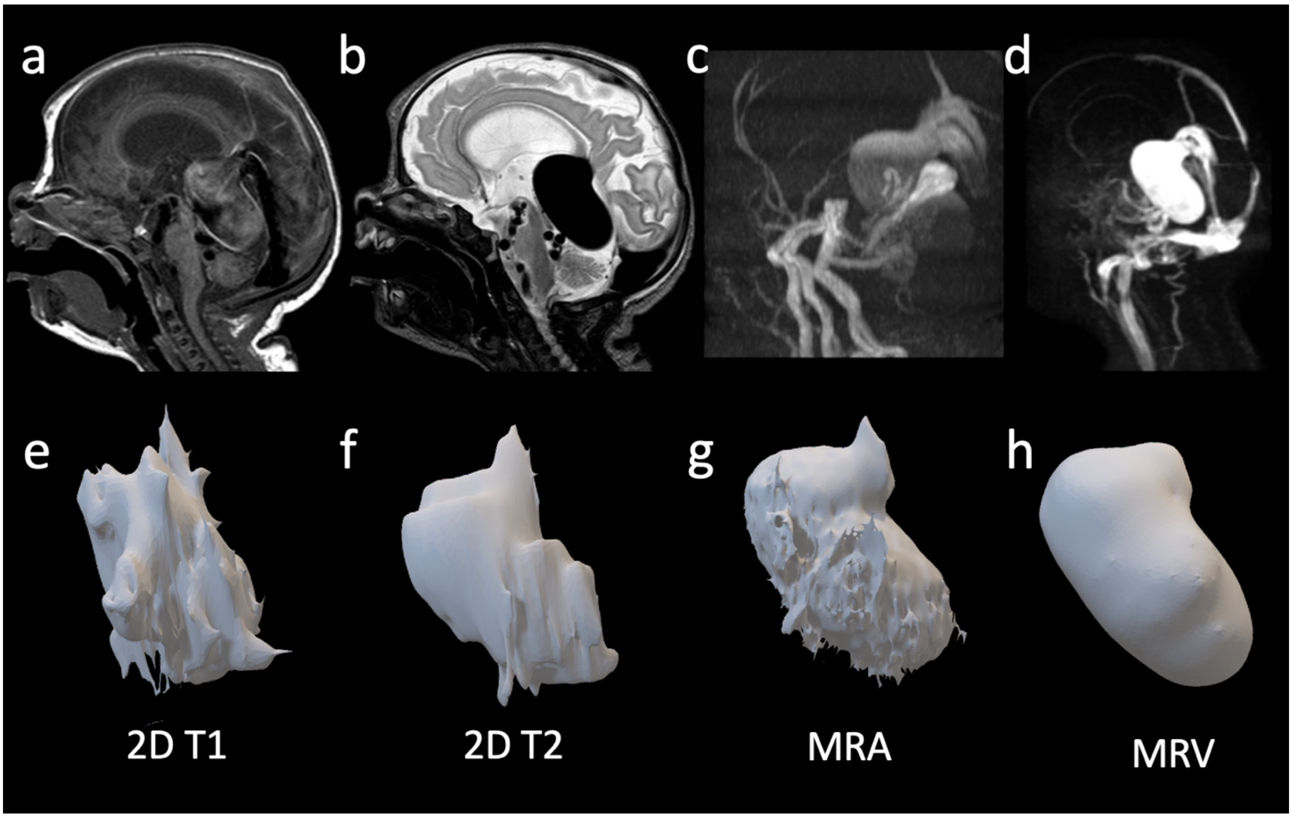

Online Resource 3

Supplementary Figure 2. Graph showing results of repeated measures of the VGAM pouch volume for the two investigated methods performed by one Reader.

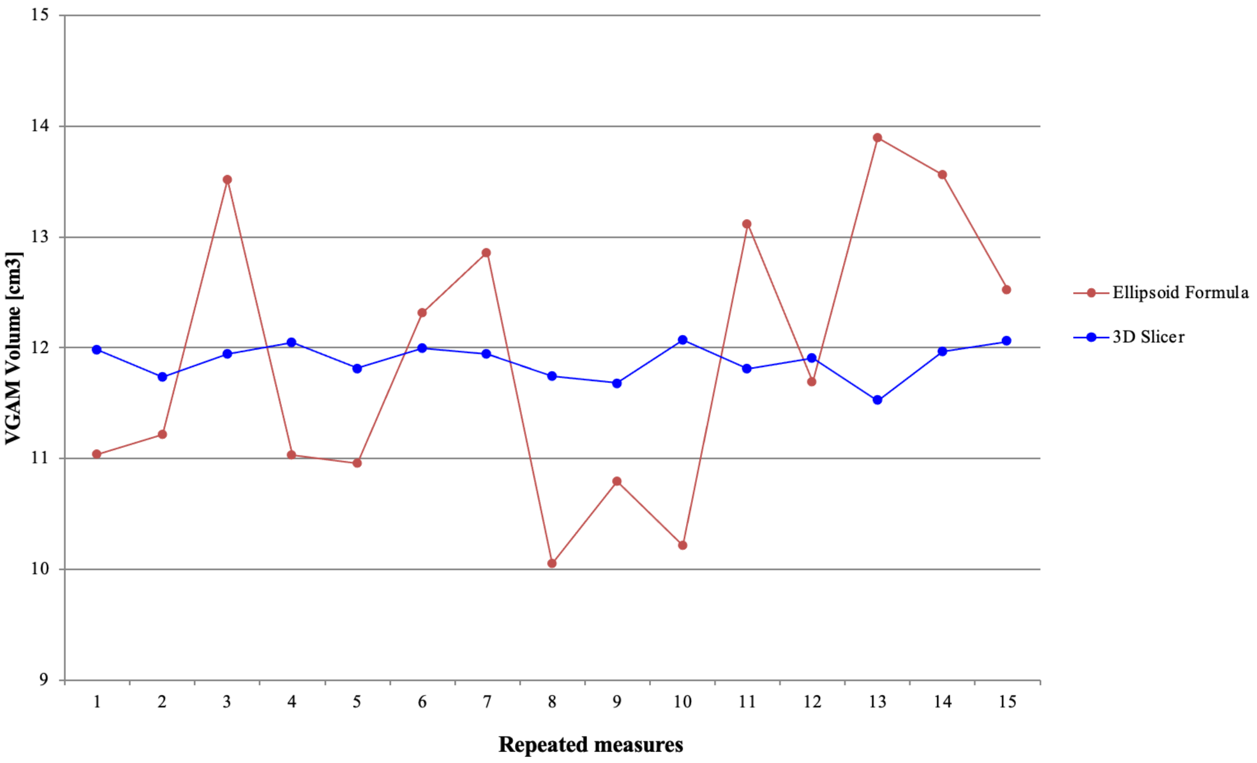

Online Resource 4

Supplementary Table 2. Description of the POPC (Paediatric Overall Performance Category) scale items

POPC category	Description
**1. Normal**	Performs at age-appropriate level in daily living activities. Medical or physical problems do not interfere with normal activity.
**2. Mild disability**	Mild medical or physical problems causing minor limitations that do not interfere with normal life (e.g., asthma). Pre-school child: may present disabilities compatible with future independent life (e.g., single amputation) and is able to perform over 75% of age-appropriate daily living activities.
**3. Moderate disability**	Medical or physical problems interfere with normal activity. Pre-school child: unable to perform some daily living activities. School-age child: able to perform multiple daily living activities but presents physical disability (e.g., cannot participate in sports competitions).
**4. Severe disability**	Pre-school child: unable to perform most daily living activities. Pre-school child: dependent for most daily living activities.
**5. Coma/vegetative state**	Unconscious. Unaware. Unable to interact verbally or cognitively with the environment.
**6. Brain death**	Brain death

Online Resource5

Supplementary Table 3. Medical treatment and intensive care support

**Pre embolization (n=39)**	**yes (%)**	**no (%)**
Epinephrine	18 (46)	21 (54)
Milrinone	18 (46)	21 (54)
Levosimendan	3 (8)	36 (92)
PGE1	5 (13)	34 (87)
Diuretic	18 (46)	21 (54)
